# Angiokeratoma of Fordyce: scrotum

**DOI:** 10.11604/pamj.2024.48.45.43647

**Published:** 2024-06-05

**Authors:** Sabiha Quazi, Keerthana Rachamadugu

**Affiliations:** 1 Department of Dermatology, Datta Meghe Medical College, Datta Meghe Institute of Higher Education and Research, Nagpur, India

**Keywords:** Angiokeratoma, scrotum, cryotherapy

## Image in medicine

Multiple, asymptomatic, firm, non-tender, skin-colored, elevated papular lesions measuring about 2-4 mm in diameter presented over the scrotal region of a 68-year-old male patient for the last 4 months. Initially, they were few, but gradually got increased in number and size over a period of time. The patient had no history of trauma, pain, itching and bleeding. On investigation, the venereal disease research laboratory (VDRL) test and human immunodeficiency virus (HIV) test were negative. A dermoscopic examination was done which showed classic well-demarcated reddish round lacunae with a whitish veil, a typical feature of angiokeratoma of Fordyce. Skin punch biopsy was also done using a 3 mm punch, which revealed hyperkeratosis and acanthosis of the epidermis with elongated rete ridges and numerous, congested and dilated thin-walled capillaries in the dermis. Based on the clinical, dermoscopic, and histopathological findings, the diagnosis of angiokeratoma of Fordyce was made. Proper counseling of the patient was done, regarding its benign nature, and the patient was treated with cryotherapy; 3 sittings were done four weeks apart.

**Figure 1:**
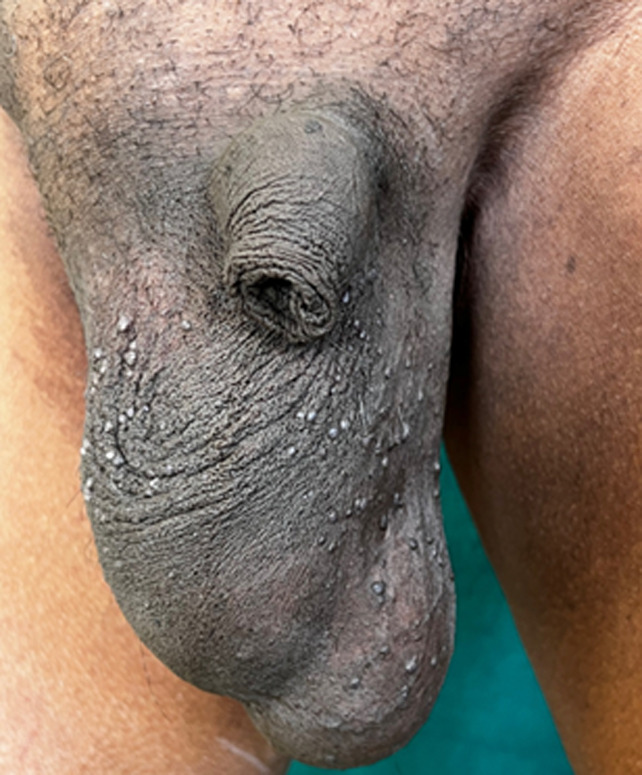
angiokeratoma of Fordyce on scrotum

